# ‘Let’s talk about sleep health’ within primary care: a qualitative study of patients’ willingness to engage in psychological interventions for insomnia

**DOI:** 10.3399/BJGP.2023.0310

**Published:** 2024-05-28

**Authors:** Brooke Swierzbiolek, Erin Oldenhof, Jamie EM Byrne, Petra K Staiger

**Affiliations:** School of Psychology, Deakin University, Burwood, Australia.; Reconnexion counsellor — Benzodiazepine Dependency Treatment Program, Reconnexion, a service of EACH; research fellow, School of Psychology, Deakin University, Burwood, Australia.; School of Psychology, Deakin University, Burwood, Australia.; School of Psychology, Deakin University, Burwood, Australia.

**Keywords:** general practice, chronic insomnia, qualitative research, theory of planned behavior, cognitive behavioral therapy, psychosocial intervention

## Abstract

**Background:**

Cognitive behavioural therapy for insomnia (CBT-I) is recommended as the first-line treatment for insomnia yet remains underutilised in general practice. Understanding patient motivations and barriers to engaging in psychological interventions for insomnia is critical. Theoretical frameworks, such as the theory of planned behaviour, are needed to identify variables related to intentions and behaviour change.

**Aim:**

To explore key influences that motivate individuals’ intention to engage with psychological interventions for insomnia.

**Design and setting:**

Qualitative study consisting of an online survey and interviews with 20 community-dwelling participants with insomnia aged 26–75 years residing in Victoria, Australia.

**Method:**

Guided by the theory of planned behaviour, reflexive thematic analysis was used to identify factors influencing participants’ intention to engage with psychological interventions for insomnia.

**Results:**

Participants reported positive attitudes towards psychological interventions for insomnia, stemming from negative beliefs about pharmacological sleep aids and the perceived benefits of a structured and evidence-based intervention. Important others positively influenced participants’ intention to engage; however, the GP influence was less consistent and often indirect. Participants believed in the efficacy of psychological interventions, but several barriers hampered their ability to benefit from them. Accessibility was identified as a key facilitator, whereas lack of knowledge and clear referral pathways were the main barriers having an impact on uptake.

**Conclusion:**

This study highlights key factors influencing patients’ intention to engage in psychological interventions for insomnia as well as opportunities for GPs to support uptake and engagement. Routine conversations about sleep health are essential to reduce the burden of untreated insomnia in the community, and the active promotion of evidence-based psychological interventions is needed.

## Introduction

Insomnia is a common health issue that presents significant treatment difficulties for GPs in primary care. Despite leading to impaired functioning across several domains (for example, reduced work productivity, social participation, and risk of motor vehicle accidents),^1^^,^^2^ treatment-seeking for insomnia remains low.^3^^,^^4^ Prevalence estimates of insomnia in adults vary widely across studies, ranging from 5% to 50%, with most data drawn from Western populations.^5^^,^^6^

Although treatment of insomnia falls within the scope of general practice, its complexity renders it difficult to treat within this context.^7^ For example, insomnia rarely occurs in isolation and is often comorbid with mental health issues or chronic health conditions.^8^^–^^10^ GPs are therefore faced with a tension between prioritising the insomnia complaint versus treating comorbid conditions, including the development of a dependency on pharmacological sleep aids (that is, benzodiazepine receptor agonists [BZRAs]). Unfortunately, patients often defer professional help until their sleep problems take on a more severe and chronic trajectory.^3^^,^^11^ Less than half of individuals will consult a healthcare professional to discuss insomnia as their primary complaint, and of those who do, the vast majority contact their GP only when symptoms have reached more severe levels or persisted for longer durations (that is, several months or years).^4^

As the primary point of contact for patients presenting with insomnia, GPs are well positioned to facilitate increased uptake of psychological interventions, given that cognitive behavioural therapy (CBT-I) is recommended as the first-line treatment approach.^12^^,^^13^ However, in most cases, individuals presenting to general practice with insomnia receive a prescription for a pharmacological sleep aid, predominantly a BZRA.^14^ Despite reported decreases in BZRA prescriptions overall, both initial and repeat prescriptions of Z-drugs (for example, zopiclone) have increased in recent years regarding insomnia management.^15^ Long-term use of BZRAs is contraindicated because of risks of dependence and other harms (for example, cognitive deficits, falls, risk of motor vehicle accidents, and worsening sleep quality).^16^^–^^19^

**Table:** How this fits in

Psychological interventions for insomnia are recommended as the first-line treatment but remain underutilised in primary care settings relative to pharmacological treatments. Coupled with known harms regarding prolonged use of benzodiazepine receptor agonists to manage insomnia, the need for increased uptake of psychological interventions is critical. This study explored the influence of key factors that motivate individuals’ intention to engage with psychological interventions, revealing the importance of active involvement of GPs in this process from the initial consultation through to supporting treatment adherence long term. By understanding the consumer perspective in conjunction with the unique clinical expertise of GPs, we have offered guidance on how to enhance patient–practitioner collaboration across the entire treatment process and increase GP confidence to facilitate increased engagement with evidence-based psychological treatment modalities.

Psychological interventions for insomnia, such as CBT-I, have been found to produce both short-term and sustained improvements in insomnia symptomology,^20^^–^^23^ and reduced reliance on pharmacological sleep aids.^24^^,^^25^ Although an effective treatment approach, a range of barriers limit their uptake in those dealing with insomnia. We argue that there needs to be greater emphasis on understanding what specific factors influence patients’ motivation and intention to engage with psychological interventions. This knowledge is critical to inform how GPs can best respond to patients presenting with sleep complaints in primary care settings.

There has been limited research that has investigated patient motivations and barriers to engaging in psychological interventions for insomnia.^26^^,^^27^ At the provider level, GPs report having poor screening processes for insomnia, limited knowledge and experience with psychological interventions, and insufficient understanding of referral pathways for insomnia.^7^^,^^28^^–^^30^ Similarly, at the patient level, limited knowledge and access have been shown to hinder the uptake of psychological interventions such as CBT-I.^28^^–^^30^ However, factors that might have an impact on patients’ intention to explore this treatment modality have received less attention and require exploration and analysis.

To better understand the putative influences on intention to engage with psychological interventions for insomnia, the utilisation of health behaviour theoretical frameworks (including the theory of planned behaviour [TPB])^31^ is warranted. The TPB posits that three key motivational influences determine an individual’s intention to engage in behaviour change:

- whether the individual is favourable or unfavourable towards the behaviour (attitudes);
- perceived social pressures to perform the behaviour (subjective norms); and
- the degree to which the individual believes that a) psychological interventions will be effective for their insomnia; and b) they have control over the outcomes of the intervention (perceived behavioural control).

Although the TPB has been extensively used to predict behaviour change and as a framework to guide intervention development across diverse domains (for example, school/work behaviour, physical activity, nutrition, sexual behaviour, and alcohol use)^32^^,^^33^ it remains underused in the context of sleep health improvement despite a recent review highlighting its utility in this area.^34^ Drawing on the TPB to facilitate a comprehensive understanding of behaviour change is important, both to guide the proposed research questions and inform the primary care response to promote increased uptake of psychological interventions for insomnia.

Therefore, the overall objective of this study was to a) explore what factors influence community-dwelling individuals’ intention to engage with psychological interventions for insomnia; and b) use this knowledge to inform the development of provider-based recommendations regarding how GPs may best facilitate their increased uptake in general practice.

To achieve these objectives, we drew on the three constructs of the TPB (attitudes, subjective norms, and perceived behavioural control):

- the first aim was to explore how attitudes regarding psychological interventions for insomnia influenced participants’ current intention to engage;
- the second aim was to explore the role (if any) of subjective norms on participants’ intention to engage with psychological interventions, specifically how important others influenced their decision to engage; and
- the third aim was to explore how participants’ confidence level and sense of control over the treatment influenced their intention, including understanding practical-level facilitators and barriers to the uptake of psychological interventions for insomnia (perceived behavioural control).

## Method

### Participants

Participants were 20 community-dwelling adults residing in Victoria, Australia, purposively sampled for heterogeneity in age, gender, and duration of sleep disturbance. Participants had expressed interest in a psychological intervention for sleep and completed an initial online questionnaire. Eligible individuals (that is, aged ≥18 years and Insomnia Severity Index score [ISI] >14, clinical level of severity)^35^ were subsequently invited via email to participate in an interview regarding their perspectives towards psychological interventions for sleep problems. Individuals with mental health comorbidities or current use of pharmacological sleep aids were not excluded from participation. The relevant institutional review board (Deakin University Human Research Ethics Committee) approved all study procedures.

### Data collection

Demographic and sleep-specific data were captured, and participants completed several validated measures of physical and mental health. The ISI provided a measure of insomnia severity.^35^ Anxiety and depressive symptomology were measured via the Generalised Anxiety Disorder (GAD-7)^36^ and Patient Health Questionnaire (PHQ-9).^37^

Interviews were conducted between September and November 2022 by a single researcher and field notes were documented from each interview. A semi-structured interview guide was developed (see Supplementary Information S1) by all authors based on the TPB and directly aligned with the study aims. Pilot testing was conducted with individuals naive to the study (aged ≥18 years) for coherence, clarity, and timing of the initial interview schedule. Feedback was collated and discussed among the research team to facilitate further refinement. The final guide elicited information relating to the core constructs of the TPB — attitudes, subjective norms, and perceived behavioural control. This enabled an exploration of the putative influences on behaviour change with respect to individuals’ intention to engage with psychological treatment, adopting the TPB as a primary framework.

Written informed consent was obtained before interviews. Interviews (mean duration 31.94 min) occurred online via a video platform (Zoom), which uses end-to-end encryption with embedded audio-recording capacity, enabling the interviewer to transcribe verbatim. All identifiers were removed from final transcripts, and participants were offered the opportunity to review and amend their transcript content as necessary. Participants received a voucher as reimbursement for their time.

### Data analysis

QSR NVivo software (release 1.7.1) was used for qualitative analysis. A mixed inductive and deductive lens to coding and thematic development was adopted. Specifically, codes were derived both from the data and TPB constructs. Braun and Clarke’s reflexive thematic analysis approach was selected as the qualitative methodology because of its theoretical flexibility.^38^^,^^39^ Coding was performed by two researchers (the first two authors) with reference to both semantic and latent features of the data. Data familiarisation entailed independent reviewing of interview transcripts followed by recursive coding of the data, meaning codes were revisited, amended, and revised as coding progressed. Researchers met to discuss code content and cluster individual codes into higher-order patterns of meaning. Candidate themes were developed and refined in an iterative process developing key themes. Regular meetings were held to ensure rigour and facilitate reflexivity. The consolidated criteria for reporting qualitative research (COREQ) guided the reporting of results.^40^

## Results

### Participants

Of 21 individuals who consented to participate, 20 proceeded to complete interviews (*n* = 1 cancelled before the interview). Participant characteristics are outlined in Table 1. Half of the sample reported long-standing insomnia (that is, >10 years), with most ISI scores falling in the moderate range (*n* = 17) and a handful in the severe range (*n* = 3) of severity (data not shown). A total of 80% (*n* = 16) of the sample had previously sought help for their sleep problem, most commonly via GP consultation (*n* = 14). The cohort further reported high levels of anxious and depressive symptomatology (Table 1). Specifically, half of participants’ scores fell in the moderate-to-severe range of anxiety (*n =* 10) and three-quarters in the moderate-to-severe range of depression (*n* = 15) (data not shown). Of note, the sample was well-educated, with 80% (*n* = 16) having attained either an undergraduate (*n* = 8) or postgraduate (*n* = 8) tertiary qualification (Table 1).

**Table 1.** Participant characteristics^a^

**Characteristic**	**Participants (*N* = 20)**
**Age, years**	
25–34	5 (25)
35–44	4 (20)
45–54	4 (20)
55–64	4 (20)
*≥*65	3 (15)

**Identified gender**	
Female	13 (65)
Male	7 (35)

**Educational attainment**	
Some high school (no certificate)	3 (15)
Year 12 equivalent	1 (5)
TAFE/diploma or degree	8 (40)
Postgraduate degree	8 (40)

**Employment**	
Part-time/full-time	16 (80)
Casual	1 (5)
Retired	1 (5)
Other (self-employed and/or student)	2 (10)

**Prior medications for sleep**	
Benzodiazepine or Z-drug	8 (40)
Antidepressant (for example, amitriptyline, doxepin, or mirtazapine)	2 (10)
Antipsychotic (for example, quetiapine)	1 (5)
Antihistamine (for example, promethazine or doxylamine succinate)	5 (25)
Melatonin	13 (65)
None	7 (35)

**Current medications for sleep**	
Benzodiazepine or Z-drug	2 (10)
Antidepressant (for example, amitriptyline, doxepin, or mirtazapine)	2 (10)
Antihistamine (for example, promethazine or doxylamine succinate)	3 (15)
Melatonin	8 (40)
None	7 (35)

**Duration of sleep problem**	
4–≤12 months	1 (5)
>1–≤2 years	2 (10)
>2–≤5 years	3 (15)
>5–≤10 years	4 (20)
>10 years	10 (50)

**ISI scores, mean (SD)**	18.5 (2.5)

**Mental health variables, mean (SD)**	
PHQ-9 scores	12.9 (3.6)
GAD-7 scores	9.2 (5.4)

a *Data are* n *(%) unless otherwise stated. GAD-7 = Generalised Anxiety Disorder-7. ISI = Insomnia Severity Index. PHQ-9 = Patient Health Questionnaire-9. SD = standard deviation. TAFE = Technical and Further Education.*

### Qualitative results

Themes identified from the interviews were mapped onto the TPB constructs of attitudes, subjective norms, and perceived behavioural control (see Box 1).

**Box 1.** Themes mapped onto the theory of planned behaviour constructs

**Behavioural intention**	**Construct**	**Themes**
**Intention to engage with a PI for insomnia**	Attitudes	Belief sleep aids have negative consequences Lack of improvement from sleep aids Perceived need for structured support Belief in strong evidence base of PIs Belief that PIs lead to long-term improvements Belief that PIs are not a quick fix Belief that PIs may not fully resolve insomnia
	Subjective norms	Important others can positively influence engagement with PIs GP hesitation to prescribe prompts patients to explore PIs GPs lack confidence and/or knowledge to actively promote PIs Societal shift in being more accepting of PIs
	Perceived behavioural control	Knowledge/insight into PIs is linked to confidence in their efficacy Prior lack of success reduces confidence in PIs Commitment and motivation influence ability to engage Personal and situational factors can impede successful engagement PI-related factors can facilitate successful engagement Ease of access supports uptake of PIs Lack of clear referral pathways hinders earlier uptake of PIs

*PI = psychological intervention.*

Attitudes focused on both positive and negative beliefs about psychological interventions for insomnia and general perceptions towards psychological interventions. Subjective norms captured the social influence of important others and the GP on individuals’ intention to engage in psychological interventions for insomnia. Perceived behavioural control reflected participants’ confidence that the psychological intervention would be effective, how much control they had over the intervention, and what influenced their uptake of the psychological intervention (that is, barriers and facilitators).

### Attitudes

Overall, participants reported favourable attitudes towards psychological interventions, reflecting an openness and willingness to engage in them, consistent with this being a treatment-seeking sample.

#### Belief sleep aids have negative consequences

Underlying the cohort’s positive attitude towards psychological interventions for insomnia was that there were minimal associated risks, unlike pharmacological sleep aids, which were perceived to have unfavourable consequences, such as dependence:

*‘Because of the dependency issues that surrounded them, they were just not something that I really wanted to go down those pathways of.’*

(Participant [P]2, duration of sleep problem >10 years)

*‘… I don’t want to become addicted.’*

(P5, duration of sleep problem >5–≤10 years)

For many, concerns around dependency stemmed from direct prior treatment-seeking experiences that influenced participants’ current intention to consider psychological interventions for insomnia:

*‘I don’t want to pop pills for the rest of my life, I don’t want to do that … if* [psychological interventions] *can help, I’m all for it.’*

(P7, duration of sleep problem >10 years)

Most of the cohort had previously used a prescription sleep aid and overwhelmingly expressed negative beliefs about their use. Almost half of these participants reported a prescription medication as the only treatment avenue explored. Several had tried melatonin with varied perceptions of its efficacy. Although some preferred it as a natural alternative, reported drawbacks included experiencing a similar hangover effect to synthetic sleep aids. Some also ‘self-medicated’ via the use of psychoactive substances (that is, alcohol and cannabis) to promote sleep.

#### Lack of improvement from sleep aids

Participants reported the lack of perceived improvement from using a range of sleep aids resulted in feelings of frustration and desperation, which further promoted a more open attitude towards psychological interventions:

*‘I’m at the point where I’m, I’m willing to try anything.’*

(P7, duration of sleep problem >10 years)

*‘… I’ve had enough of … not getting quality sleep, and I’m at the stage where I need to do something, um, so … I’m going to do everything in my power to make sure that I follow them* [psychological interventions]*.’*

(P2, duration of sleep problem >10 years)

#### Perceived need for structured support

Overwhelmingly, participants perceived a need for structured professional guidance*,* support, and accountability to assist their efforts to improve sleep. Despite several participants’ awareness and practice of sleep hygiene strategies, there was recognition that they could benefit from additional knowledge and support in this practice:

*‘… I’m aware-, I’ve read a bit about sleep hygiene … I didn’t find that help-, has helped me much.’*

(P19, duration of sleep problem >5–≤10 years)

*‘I just think having some guidance and professional skills, that’s what I’m looking for … like I just need strategies … and a therapy that I can follow.’*

(P4, duration of sleep problem >10 years)

#### Belief in strong evidence base of psychological interventions for insomnia.

Participants further expressed that knowing psychological interventions have a strong evidence base enhanced their willingness to consider this treatment modality to resolve their insomnia:

*‘… I think specifically because it’s evidence-based as well … I feel like that kind of gives it like a sense of like credibility to it, and that I feel more inclined to, like, believe in the efficacy of it.’*

(P8, duration of sleep problem >2–≤5 years)

#### Belief that psychological interventions lead to long-term improvements

Importantly, psychological interventions were considered to lead to long-term sustainable sleep outcomes, resulting in a generalised increase in wellbeing with transferable benefits to other areas of life:

*‘I think it could … be advantageous to like other aspects of life, mental health in general, and, um, even anxiety … it’s all, inter-, interlinked.’*

(P15, duration of sleep problem >2–≤5 years)

#### Belief that psychological interventions for insomnia are not a quick fix

Although there were several reported negative beliefs regarding psychological interventions, participants indicated these did not affect their current intention to engage. The predominant negative beliefs were that psychological interventions required time, effort, and commitment to be effective:

*‘I would say they are not an immediate quick fix, it’s often something that I guess takes time … they are often to do with a practice, so it’s something you need to continually work on and implement. It’s not as easy as taking a tablet … it takes work.’*

(P12, duration of sleep problem >5–≤10 years)

#### Belief that psychological interventions may not fully resolve insomnia

Some also expressed concern that psychological interventions would not completely resolve their condition, and others were concerned that overreliance or rigid use of psychological strategies might lead to reduced treatment effectiveness:

*‘Um, I’m confident that it will have some impact. I’m not confident that it will completely solve the issue.’*

(P13, duration of sleep problem >10 years)

*‘Overuse of them* [psychological strategies] *sometimes is a disadvantage … like they’re successful, and it’s like, you keep using them and using them and then all of a sudden … they don’t become useful anymore.’*

(P2, duration of sleep problem >10 years)

### Subjective norms

#### Important others can positively influence engagement with psychological interventions for insomnia

Most individuals perceived that psychological interventions for insomnia were viewed favourably by important others (that is, family or close friends):

*‘I know one hundred per cent that everyone would be supportive. Everybody wants to know. I’ve told people, they’re like “as soon as you find out, can you please tell me?”’*

(P4, duration of sleep problem >10 years)

Indeed, several participants reported that important others had actively promoted this treatment avenue:

*‘It was my husband that sent me the Facebook feed through … to say, “why don’t you, why don’t you try that?”’*

(P1, duration of sleep problem 4–≤12 months)

#### GP hesitation to prescribe prompts patients to explore psychological interventions for insomnia

The GP influence tended to be indirect, resulting from caution or an unwillingness to prescribe pharmacological sleep aids long term, leaving participants feeling the need to explore other avenues:

*‘When I ask for Imovane, he* [GP] *says “oh, you know, I’ll prescribe it now, but, um, maybe we need to talk about, um, sleep health and that sort of thing”.’*

(P20, duration of sleep problem >5–≤10 years)

Although some GPs were collaborative and patient centred in their approach, other participants reported that their GP was dismissive of their experience, neither willing to prescribe a pharmacological sleep aid nor able to support alternative treatments:

*‘…* [GPs] *would flat out refuse and said, “no, we don’t do that … you need to try other things” … and that’s sort of the end of the conversation. They say try other things, but there’s no, um, no like, you know, “go here” or “do this” or “this program’s great”.’*

(P9, duration of sleep problem >10 years)

#### GPs lack confidence and/or knowledge to actively promote psychological interventions for insomnia

In some cases, GPs promoted generalised sleep hygiene strategies, yet only one participant reported being informed about more comprehensive interventions (that is, CBT-I). Almost half of the participants believed that their GP lacked sufficient knowledge to offer psychological interventions (and therefore relied on pharmacological sleep aids) and perceived GP confidence to promote psychological interventions for insomnia as low:

*‘The doctor said, “oh well, just take some more, take some more, take some more” — it’s not right. It’s not, not fixing the problem, it’s only masking it.’*

(P17, duration of sleep problem >10 years)

*‘I just don’t know if they* [GPs] *have the experience to have been able to offer me anything else for sleep besides what they already had.’*

(P2, duration of sleep problem >10 years)

#### Societal shift in being more accepting of psychological interventions for insomnia

Lastly, several participants, primarily males, reported a societal shift in attitudes towards seeking psychological support. For these participants, perceived reductions in stigma and normalisation of help-seeking underpinned their current intention to engage:

*‘Um, but I’m seeing a lot of positive change in, in society now. It’s becoming a lot more receptive and more open to accepting, ah, psychological assistance.’*

(P14, duration of sleep problem >1–≤2 years)

*‘I don’t think there’s a stigma around psychological intervention for sleep.’*

(P18, duration of sleep problem >10 years)

### Perceived behavioural control

#### Knowledge and/or insight into psychological interventions for insomnia is linked to confidence in their efficacy

In general, participants expressed moderate-to-high levels of confidence that psychological interventions would be effective, increasing their willingness to engage. Higher levels of confidence were more evident in participants who possessed knowledge about psychological interventions more broadly or perceived their insomnia to originate from cognitive or behavioural factors:

*‘I think they* [psychological interventions] *probably could help a lot because I’m sure it’s all, a lot of it’s in my head, my mind.’*

(P5, duration of sleep problem >5–≤10 years)

#### Prior lack of success reduces confidence in psychological interventions for insomnia

In contrast, moderate levels of confidence were more evident in those who either had unsuccessful prior treatment-seeking experiences with pharmacological sleep aids, lacked knowledge of what to expect from treatment, or stemmed from self-preservation (that is, not wanting to get their hopes up):

*‘If I hadn’t tried a million other things before then, you know, maybe my confidence would be up.’*

(P16, duration of sleep problem >10 years)

#### Commitment and motivation influence ability to engage

Although participants almost universally recognised that outcomes related to engaging with psychological interventions were self-determined, therefore under their control, they noted a range of barriers and facilitators. Many participants recognised that their degree of commitment, consistency, and motivation to engage could be either a barrier or facilitator influencing the success of the intervention.

#### Personal and situational factors can impede successful engagement

Specific barriers reported at the individual level that could impede successful adherence to a psychological intervention were energy levels, time, mood, and stress:

*‘My own motivation, that’s more where my concerns come in, it’s not so much psychological interventions that I’m concerned about, it’s more me following any plans that get put into place … ’*

(P2, duration of sleep problem >10 years)

*‘It is having the time, but also the energy to be able to commit and implement and follow through … the energy is then linked to the poor sleep. So, that’s a kind of a whole cyclic problem in and of itself.’*

(P12, duration of sleep problem >5–≤10 years)

Participants further anticipated employment (for example, travel and shift work) and sleep environment (for example, disruptions attributable to environmental noise, children, or pets) as external barriers to successfully engage with psychological interventions:

*‘Things happening at home stops me from focusing on carrying out the treatment … it’s kind of hard to block out the environmental sort of things.’*

(P8, duration of sleep problem >2–≤5 years)

#### Psychological intervention-related factors can facilitate successful engagement

In terms of facilitators, most participants reported that psychological interventions needed to provide certain qualities to be effective. These included the need for an evidence-based treatment, a collaborative and consultative therapeutic relationship, and an intervention that was both personalised and delivered in a manner that facilitated understanding of the content.

#### Ease of access supports uptake of psychological interventions for insomnia

Participants described several barriers and facilitators having an impact on their uptake of the psychological intervention in the first place. Facilitators all related to accessibility, namely that the intervention was delivered online, did not require referral or assessment, there were little to no wait times, and incurred no cost:

*‘… to go through the, the loops of, um, like mental health plans at the sleep clinics, there’s waiting lists, I felt like this* [psychological intervention] *was great because it was straight away.’*

(P1, duration of sleep problem 4–≤12 months)

#### Lack of clear referral pathways hinders earlier uptake of psychological interventions for insomnia

Barriers to earlier uptake of a psychological intervention reflected a lack of clear treatment pathways, where participants reported being overwhelmed by the information available online, lacking guidance from GPs and sleep specialists, and limited understanding about psychological interventions and insomnia more broadly:

*‘… prior to today, I didn’t think they* [psychological interventions] *were relevant to me, um, because I don’t have any trouble falling asleep.’*

(P10, duration of sleep problem >2–≤5 years)

*‘… it’s* [psychological interventions] *a new avenue I hadn’t really considered or, um, heard of.’*

(P14, duration of sleep problem >1–≤2 years)

## Discussion

### Summary

Findings from this study indicate that participants’ favourable attitudes towards psychological interventions were primarily related to dissatisfaction with prior treatment-seeking experiences (that is, pharmacological sleep aids). Our sample was treatment-seeking for chronic insomnia, which, in addition to prompting feelings of frustration and desperation, may have culminated in their expressed need for structured professional guidance and evidence-based psychological interventions to improve their sleep.

The positive influence of family and friends on engaging with a psychological intervention was encouraging and, in many cases, appeared to be the impetus for trying something new. In contrast, the GP influence was less clear or consistent in promoting intention to engage, highlighting the need for more active GP involvement in managing insomnia in primary care.

Lastly, participants believed psychological interventions would effectively treat their insomnia, particularly if they perceived their sleep issue had psychological or behavioural underpinnings. They were realistic about their ability to adhere to the intervention, recognising the impact of their own motivation and commitment, as well as situational influences. It appeared that ease of access was a key facilitator that saw participants enrol in a psychological intervention, whereas lack of support, information, and clear referral pathways impeded earlier engagement.

### Strengths and limitations

To the authors’ knowledge, this is the first study to apply a behaviour change theoretical framework to individuals’ lived experience of insomnia to better understand what factors influence intention to engage with psychological interventions. This work importantly addresses recent calls for greater utilisation of health behaviour change theoretical frameworks to support sleep health improvement efforts.^34^ The sample included a range of demographics (that is, varying in age and gender, psychiatric/physical comorbidities, degree of insomnia severity and chronicity, and use of pharmacological sleep aids). Therefore, the provider-based recommendations developed have wide-reaching relevance to many subgroups with insomnia. Several limitations are also important to note.

First, sample characteristics, including a mostly White and university-educated community sample, may limit generalisability to other cultural or minority groups. Education levels may be important in understanding treatment uptake, adherence, and responses to psychological interventions for mental health conditions more broadly (for example, CBT for depression) and thus are an important avenue for future research. Second, given that we recruited participants who had expressed interest in a psychological intervention for insomnia, attitudes and intentions expressed by our cohort may not be broadly reflective of all individuals with insomnia, particularly non-treatment-seeking segments of the population with insomnia. Lastly, this study explored individuals’ intention to engage as a proxy for engagement, which may not directly map onto actual uptake (that is, participants had registered to participate but were yet to actively engage).

### Comparison with existing literature

Although the TPB theoretical framework has been adopted to understand intention to engage in various health behaviours^33^^,^^41^ it remains underutilised in insomnia research to date.^34^ Given that its utility lies in its ability to identify the key factors underlying intention to engage in behaviour change (that is, attitudes, subjective norms, and perceived behavioural control),^31^ we envisage that active translation of current findings will contribute to meaningful improvements in how insomnia is managed within medical care and health settings more broadly.

Health professionals themselves have identified limited knowledge and experience with CBT-I and insufficient understanding of referral pathways for insomnia as barriers to initiating referrals for chronic sleep problems.^7^^,^^28^^–^^30^ These are mirrored by current findings, whereby participants perceived GPs lacked confidence and requisite knowledge to actively promote psychological interventions for insomnia. Of note, only one participant reported being informed about more comprehensive interventions (that is, CBT-I from their GP). Although sleep hygiene education is not endorsed as an effective stand-alone treatment for insomnia,^42^ qualitative research suggests most GPs tend to rely on it as a first-line treatment option, with some even considering it a means to avoid prescription of pharmacological sleep aids (for example, BZRAs).^7^ As echoed in our cohort, several participants referenced their GP’s unwillingness to prescribe and despite adherence to sleep hygiene recommendations had derived limited benefit from this approach alone. Taken together, GPs would likely benefit from additional training to facilitate increased awareness and understanding of evidence-based psychological interventions for insomnia, including CBT-I, to support optimal patient outcomes.

Moreover, when considering management strategies for patients with insomnia, GPs reported they would consider referring patients with presentations of anxiety and depression to psychological services yet did not perceive these same interventions to be available for insomnia management.^43^ Indeed, insomnia complaints were rarely considered the primary issue and more often attributed to an underlying physical health or mental health condition,^43^ which is noteworthy given high rates of comorbid depressive and anxious symptomatology in our cohort. Greater GP awareness of insomnia as a primary condition, not simply a symptom of a comorbid condition, is critical in primary care settings, given its implications for early identification of insomnia and, therefore, earlier access to effective treatments.

Lastly, although scarce, research on patients’ decision-making processes for managing insomnia revealed that patients’ treatment beliefs, external social influences (that is, advice from family/friends and healthcare professionals), and prior treatment-seeking experiences were linked to patients’ preference for non-pharmacological interventions, rather than pharmacological interventions.^44^ Although the current study identified similar factors influencing participants’ preference for psychological intervention, a limitation of earlier work was that patient preferences were based on hypothetical treatment scenarios. The present study advances understanding to suggest that treatment beliefs, social influences, and prior treatment-seeking experiences are linked not only to treatment preferences^44^ but also to intention to engage and preliminary steps towards the uptake of psychological interventions (that is, participants in the current study had registered to take part in a psychological intervention for insomnia).

### Implications for practice

Findings from this study have informed the development of a range of provider-based recommendations to support GPs in their work (see Box 2 for a complete list). These recommendations were guided by the influence of attitudes, subjective norms, and participants’ perceived behavioural control to engage with psychological interventions to ultimately bridge the gap between intention to engage and actual uptake and engagement.

**Box 2.** Provider-based recommendations to support engagement with CBT-I

**Stage of client readiness**	**Provider recommendations**
Non-treatment-seeking for insomnia: increase patient receptiveness to psychological interventions	Prioritise engagement and rapport-building Routinely initiate conversations about sleep (including psychoeducation about insomnia and screening for sleep disorders) Motivational interviewing to assess and promote readiness to engage in treatment Raise awareness regarding the impact of untreated insomnia Education about short-term and intermittent use of BZRAs
Early treatment-seeking for insomnia: increase intention to engage with psychological interventions	Raise awareness and explore impact of untreated insomnia Education about short-term and intermittent use of BZRAs Promote effectiveness of psychological interventions (that is, CBT-I) for sleep and mental wellbeing Motivational interviewing to support uptake of psychological interventions for insomnia Refer to online evidence-based programmes with low or no associated patient cost Refer to stepped-care treatments where possible
Patient engaged with CBT-I: increase/maintain adherence to psychological interventions	Regularly review progress and impact on insomnia and mental wellbeing Reinforce the effectiveness of evidence-based treatments such as CBT-I Motivational interviewing to increase or maintain adherence to psychological interventions Shared-care approach: collaborate with sleep health practitioners and patients to support optimal treatment outcomes

*BZRA = benzodiazepine receptor agonists. CBT-I = cognitive behavioural therapy for insomnia.*

Given the cumulative harms and chronicity of insomnia, GPs need additional training and skills to routinely screen for and educate patients on insomnia and psychological treatment options. As seen in some participants in our study, patients may further lack insight into psychological contributors to their sleep problems, which may lead to developing more intractable sleep problems or ongoing use of pharmacological sleep aids. GPs initiating conversations about sleep difficulties, and patients' perceptions of the underlying cause of their sleep problem (biological or psychological origins) as routine practice are essential steps towards greater uptake and engagement with psychological interventions for insomnia. Educating patients on the impact of untreated insomnia and the availability of evidence-based, efficacious treatments such as CBT-I also constitute key intervention points for GPs.

GPs could also consider stepped-care approaches in their approach to insomnia management, given that they accommodate diverse individual needs and allow treatment to be tailored to match each patient’s preferences.^27^^,^^44^ Lower-level steps may involve referrals to digital CBT-I programmes if available (for example, Sleepio and This Way Up).^45^^–^^48^ Although access to locally based digital CBT-I programmes with demonstrated effectiveness remains challenging, they hold promise as a cost-effective and scalable option to support insomnia management in community settings. For individuals using a BZRA as a pharmacological sleep aid, a stage-based approach to deprescribing is recommended^49^ while facilitating engagement with psychological interventions (that is, CBT-I) to support long-term insomnia management.^50^^,^^51^ Lastly, current findings further suggest normalisation of psychological help-seeking as particularly important when consulting with male patients, given that they may be more sensitive to self-stigma associated with traditional masculinity norms.^52^

Beyond initial identification of insomnia, patients are also likely to benefit from GPs’ continued support and monitoring throughout their engagement with psychological interventions. Given the range of factors that may influence a person’s ability to adhere to psychological interventions, GPs may adopt the role of a ‘coach’ to support optimal treatment outcomes, particularly where patients are concurrently engaged in BZRA deprescribing.^53^ Moreover, even one-time sleep consultations involving the GP, patient, and a sleep specialist can have a notable impact on GPs’ attention towards sleep issues and lead to improvements in patients’ sleep.^54^ Given time constraints inherent to general practice, these may be feasible approaches to support patients’ ongoing insomnia management.

Ultimately, insomnia is a significant health issue that needs to be proactively addressed in the primary care setting but it also warrants treatment at the population level. Increased provision of GP resources regarding insomnia assessment, management, and referral pathways are needed to support GPs to enact these recommendations, given time constraints inherent in primary care settings and the degree to which GPs are presently underresourced and overworked. Increased uptake and engagement with psychological interventions for insomnia is contingent on a collaborative approach to care that involves the patient, treatment providers (both GPs and sleep specialists), and input from overarching systems.

Of concern, individuals with insomnia often endure chronic poor sleep and subsequent physical and mental health problems before accessing adequate treatment. For GPs, insomnia often presents complexities that are challenging to manage in the primary care setting.^7^ Nonetheless, GPs can play a pivotal role in supporting patient engagement with psychological interventions. Current findings demonstrate that individuals are open and willing to engage with psychological interventions, but active involvement of GPs is critical to support earlier uptake. Guided by the TPB as a theoretical framework, key provider-based recommendations were developed to increase GP confidence to promote psychological interventions for insomnia. Population-level efforts need to prioritise treatment development and implementation of low-cost psychological interventions to support GPs in this work. This study further highlights the usefulness of health behaviour change models to support increased uptake of psychological interventions for insomnia, and their continued use in sleep research is encouraged.

## References

[1] Daley M, Morin CM, LeBlanc M, Grégoire J-P (2009). The economic burden of insomnia: direct and indirect costs for individuals with insomnia syndrome, insomnia symptoms, and good sleepers. Sleep.

[2] Chung KF, Kwok CW, Yeung WF (2018). Predictors of daytime consequences of insomnia: th e roles of quantitative criteria and nonrestorative sleep. Psychopathology.

[3] Aikens JE, Rouse ME (2005). Help-seeking for insomnia among adult patients in primary care. J Am Board Fam Pract.

[4] Torrens Darder I, Argüelles-Vázquez R, Lorente-Montalvo P (2021). Primary care is the frontline for help-seeking insomnia patients. Eur J Gen Pract.

[5] Ohayon MM (2002). Epidemiology of insomnia: what we know and what we still need to learn. Sleep Med Rev.

[6] Morin CM, Jarrin DC (2022). Epidemiology of insomnia: prevalence, course, risk factors, and public health burden. Sleep Med Clin.

[7] Haycock J, Grivell N, Redman A (2021). Primary care management of chronic insomnia: a qualitative analysis of the attitudes and experiences of Australian general practitioners. BMC Fam Pract.

[8] Fernandez-Mendoza J, Vgontzas AN (2013). Insomnia and its impact on physical and mental health. Curr Psychiatry Rep.

[9] Grandner MA (2014). Addressing sleep disturbances: an opportunity to prevent cardiometabolic disease?. Int Rev Psychiatry.

[10] Morin CM, Benca R (2012). Chronic insomnia. Lancet.

[11] Henry D, Rosenthal L, Dedrick D (2013). Understanding patient responses to insomnia. Behav Sleep Med.

[12] Qaseem A, Kansagara D, Forciea MA (2016). Management of chronic insomnia disorder in adults: a clinical practice guideline from the American College of Physicians. Ann Intern Med.

[13] Riemann D, Baglioni C, Bassetti C (2017). European guideline for the diagnosis and treatment of insomnia. J Sleep Res.

[14] Miller CB, Valenti L, Harrison CM (2017). Time trends in the family physician management of insomnia: the Australian experience (2000–2015). J Clin Sleep Med.

[15] Begum M, Gonzalez-Chica D, Bernardo C (2021). Trends in the prescription of drugs used for insomnia: an open-cohort study in Australian general practice, 2011–2018. Br J Gen Pract.

[16] Brandt J, Leong C (2017). Benzodiazepines and z-drugs: an updated review of major adverse outcomes reported on in epidemiologic research. Drugs R D.

[17] Kaufmann CN, Spira AP, Depp CA, Mojtabai R (2016). Continuing versus new prescriptions for sedative-hypnotic medications: United States, 2005–2012. Am J Public Health.

[18] McGee N, Proctor JL, Hart AM, Burman M (2021). Reconsidering benzodiazepines and z-drug prescriptions: responsible prescribing and deprescribing. J Nurse Pract.

[19] Tannenbaum C, Paquette A, Hilmer S (2012). A systematic review of amnestic and non-amnestic mild cognitive impairment induced by anticholinergic, antihistamine, GABAergic and opioid drugs. Drugs Aging.

[20] Trauer JM, Qian MY, Doyle JS (2015). Cognitive behavioral therapy for chronic insomnia: a systematic review and meta-analysis. Ann Intern Med.

[21] Wu JQ, Appleman ER, Salazar RD, Ong JC (2015). Cognitive behavioral therapy for insomnia comorbid with psychiatric and medical conditions: a meta-analysis. JAMA Intern Med.

[22] van Straten A, van der Zweerde T, Kleiboer A (2018). Cognitive and behavioral therapies in the treatment of insomnia: a meta-analysis. Sleep Med Rev.

[23] van der Zweerde T, Bisdounis L, Kyle SD (2019). Cognitive behavioral therapy for insomnia: a meta-analysis of long-term effects in controlled studies. Sleep Med Rev.

[24] Blom K, Rück C, Lindefors N (2016). Three-year follow-up of insomnia and hypnotics after controlled internet treatment for insomnia. Sleep.

[25] Park KM, Kim TH, An SK (2018). Cognitive behavioral therapy for insomnia reduces hypnotic prescriptions. Psychiatry Investig.

[26] Koffel E, Amundson E, Polusny G, Wisdom JP (2020). “You’re missing out on something great”: patient and provider perspectives on increasing the use of cognitive behavioral therapy for insomnia”. Behav Sleep Med.

[27] Koffel E, Branson M, Amundson E, Wisdom JP (2021). “Sign me up, I’m ready!”: helping patients prescribed sleeping medication engage with cognitive behavioral therapy for insomnia (CBT-I)”. Behav Sleep Med.

[28] Araújo T, Jarrin DC, Leanza Y (2017). Qualitative studies of insomnia: current state of knowledge in the field. Sleep Med Rev.

[29] Cheung JY, Bartlett D, Armour C (2014). Insomnia patients’ help-seeking experiences. Behav Sleep Med.

[30] Koffel E, Bramoweth AD, Ulmer CS (2018). Increasing access to and utilization of cognitive behavioral therapy for insomnia (CBT-I): a narrative review. J Gen Intern Med.

[31] Ajzen I (1991). The theory of planned behavior. Organ Behav Hum Decis Process.

[32] Steinmetz H, Knappstein M, Ajzen I (2016). How effective are behavior change interventions based on the theory of planned behavior? A three-level meta-analysis. Z Psychol.

[33] McEachan RRC, Conner M, Taylor NJ, Lawton RJ (2011). Prospective prediction of health-related behaviours with the theory of planned behaviour: a meta-analysis. Health Psychol Rev.

[34] Mead MP, Irish LA (2020). Application of health behaviour theory to sleep health improvement. J Sleep Res.

[35] Morin CM, Belleville G, Bélanger L, Ivers H (2011). The Insomnia Severity Index: psychometric indicators to detect insomnia cases and evaluate treatment response. Sleep.

[36] Spitzer RL, Kroenke K, Williams JBW, Löwe B (2006). A brief measure for assessing generalized anxiety disorder: the GAD-7. Arch Intern Med.

[37] Kroenke K, Spitzer RL, Williams JB (2001). The PHQ-9: validity of a brief depression severity measure. J Gen Intern Med.

[38] Braun V, Clarke V (2006). Using thematic analysis in psychology. Qual Res Psychol.

[39] Braun V, Clarke V (2021). One size fits all? What counts as quality practice in (reflexive) thematic analysis?. Qual Res Psychol.

[40] Tong A, Sainsbury P, Craig J (2007). Consolidated criteria for reporting qualitative research (COREQ): a 32-item checklist for interviews and focus groups. Int J Qual Health Care.

[41] McInnes C, Carstairs SA, Cecil JE (2023). A qualitative study of young peoples’ thoughts and attitudes to follow a more plant-based diet. Front Psychol.

[42] Edinger JD, Arnedt JT, Bertisch SM (2021). Behavioral and psychological treatments for chronic insomnia disorder in adults: an American Academy of Sleep Medicine systematic review, meta-analysis, and GRADE assessment. J Clin Sleep Med.

[43] Everitt H, McDermott L, Leydon G (2014). GPs’ management strategies for patients with insomnia: a survey and qualitative interview study. Br J Gen Pract.

[44] Cheung JMY, Bartlett DJ, Armour CL (2018). To drug or not to drug: a qualitative study of patients’ decision-making processes for managing insomnia. Behav Sleep Med.

[45] Sweetman A, Knieriemen A, Hoon E (2021). Implementation of a digital cognitive behavioral therapy for insomnia pathway in primary care. Contemp Clin Trials.

[46] Espie CA, Kyle SD, Williams C (2012). A randomized, placebo-controlled trial of online cognitive behavioral therapy for chronic insomnia disorder delivered via an automated media-rich web application. Sleep.

[47] Espie CA, Emsley R, Kyle SD (2019). Effect of digital cognitive behavioral therapy for insomnia on health, psychological well-being, and sleep-related quality of life: a randomized clinical trial. JAMA Psychiatry.

[48] Grierson AB, Hobbs MJ, Mason EC (2020). Self-guided online cognitive behavioural therapy for insomnia: a naturalistic evaluation in patients with potential psychiatric comorbidities. J Affect Disord.

[49] Oldenhof E, Mason T, Anderson-Wurf J, Staiger PK (2021). Role of the prescriber in supporting patients to discontinue benzodiazepines: a qualitative study. Br J Gen Pract.

[50] Lee JY, Farrell B, Holbrook AM (2019). Deprescribing benzodiazepine receptor agonists taken for insomnia: a review and key messages from practice guidelines. Pol Arch Intern Med.

[51] Hintze JP, Edinger JD (2022). Hypnotic discontinuation in chronic insomnia. Sleep Med Clin.

[52] Yousaf O, Popat A, Hunter MS (2015). An investigation of masculinity attitudes, gender, and attitudes toward psychological help-seeking. Psychol Men Masc.

[53] Coteur K, Van Nuland M, Schoenmakers B (2023). Implementing blended care to discontinue benzodiazepine receptor agonist use for insomnia: process evaluation of a pragmatic cluster randomized controlled trial. JMIR Form Res.

[54] Edinger JD, Grubber J, Ulmer C (2016). A collaborative paradigm for improving management of sleep disorders in primary care: a randomized clinical trial. Sleep.

